# Clinical outcome after lipectomy in the management of patients with human immunodeficiency virus-associated dorsocervical fat accumulation: An observational cohort study

**DOI:** 10.1097/MD.0000000000016112

**Published:** 2019-06-21

**Authors:** Song Chen, Xi-en Gui, Qian Cao, Jean-Pierre Routy

**Affiliations:** a Department of Urology; b Department of Biological Repositories; c Training Center of AIDS Prevention and Cure of Hubei Province; d Department of Infectious Diseases, Zhongnan Hospital of Wuhan University, Wuhan, China; e Division of Hematology, and Chronic Viral Illness Service, Research Institute of the McGill University Health Center, Montreal, Quebec, Canada.

**Keywords:** buffalo hump, human immunodeficiency virus, lipectomy, lipodystrophy

## Abstract

Lipo-accumulation of the dorsocervical fat pad (“buffalo hump”) is a complication observed in people living with human immunodeficiency virus (HIV). We described the clinical outcome of people living with HIV with “buffalo hump” treated by excisional lipectomy.

From April 2013 to March 2018, medical records of people living with HIV, who received care in our hospital have been evaluated. Among them, patients with dorsocervical fat accumulation treated by excisional lipectomy have been retrospectively assessed.

Nine patients with “buffalo hump” among 2886 people living with HIV (3.1‰, 9/2886) were included. Eight were women with a mean age of 47.9 ± 8.0 years old (range, 36–60). Most of them have been infected by blood transfusion (77%, 7/9) and the mean duration of HIV infection was 14.1 ± 5.5 years (range, 6–22). The mean duration for antiretroviral therapy was 8.8 ± 2.1 years (range, 6–11). The mean pre-ART CD4+ T cell count was 91.3 ± 76.5 cells/μL (range, 4–233) and 477.4 ± 271.8 cells/μL (range, 114–926) at the time of surgery. All 9 patients underwent excisional lipectomy of their hypertrophied dorsocervical fat pad. The mean size of the excised specimens was 14 × 11 × 6 cm. The median follow-up time was 24 months (range, 2–60), all 9 patients reported satisfaction with their results, with no recurrence has been observed.

Corrective surgery used to treat localized fat accumulations in people living with HIV with “buffalo hump” showed a favorable effect and can therefore be considered when necessary. Whereas drugs such as integrase inhibitors may avoid lipo-accumulation related syndrome and should be given to people living with HIV in China.

## 1 Introduction

Life of persons living with human immunodeficiency virus (HIV) has improved since the introduction of antiretroviral therapy (ART). Nevertheless, as these individuals are receiving ART for long-term, complications are increasingly reported, including the HIV-associated lipodystrophy syndrome (HALS), which is characterized by abnormal fat metabolism and tissue deposition. Several characteristics, such as older age, white race, low body weight, low nadir CD4+ T cell count, long duration of ART and exposure of protease inhibitors (PIs), or some nucleoside reverse transcriptase inhibitors (NRTIs) have been identified as risk factors for HALS.^[1]^ This syndrome is also associated with metabolic abnormalities such as dyslipidemia, insulin resistance, and glucose intolerance. The prevalence of HALS in patients on ART has been reported to be 10% up to 40%.^[2,3]^ Areas of adipose hypertrophy or lipo-accumulation (LA) include the dorsocervical region (“buffalo hump”), the anterior neck, the upper torso, and the abdomen, whereas lipoatrophy occurs in the face and the limbs. In severe cases, LA can be disfiguring, which can cause stigma and discrimination, leading to risks of poor adherence, medication discontinuation, and treatment failure.^[4]^ Surgical or cosmetic corrective treatment is the only intervention with almost immediate esthetical result, as well as the change of classes of ART when possible. Herein, we described the clinical manifestations and treatment outcome in HIV-infected patients with dorsocervical fat accumulation treated by excisional lipectomy in 1 Chinese clinical center.

## 2 Materials and methods

### 2.1 Patient characteristics and data collection

Zhongnan Hospital of Wuhan University is the largest referral hospital for HIV/AIDS care in Hubei Province, central China. From April 2013 to March 2018, medical records of people living with HIV (PLWH) who received care in our hospital have been evaluated. Among them, we identified 9 patients presenting with dorsocervical fat accumulation treated by excisional lipectomy. Age, sex, weight, height, body mass index (BMI), duration of HIV infection, mode of HIV acquisition, CD4+ T cell count, ART regimens, serum levels of total cholesterol (TC), triglycerides (TG), low density lipoprotein cholesterol (LDL-C), high density lipoprotein cholesterol (HDL-C), and fasting blood glucose (FBG) were collected. Evaluation of liver steatosis was assessed by echography or computed tomography (CT). Patients information on coinfection with hepatitis B virus (HBV), hepatitis C virus (HCV), and human papillomavirus (HPV) were also collected.

### 2.2 Ethical consideration

This clinical study was conducted according to the principles expressed in the Declaration of Helsinki. The Ethical approval was granted by the Ethics Committee of Zhongnan Hospital of Wuhan University (NO.2018055).

### 2.3 Statistical analyses

Continuous variables were described as means, medians, and ranges. We used SPSS 16.0 (Chicago, IL) to perform all statistical analyses.

## 3 Results

### 3.1 Characteristics of study participants

From a total of 2886 PLWH (2061 men and 825 women, sex ratio was 2.5:1) hospitalized in our center between April 2013 and March 2018, 9 of them had dorsocervical fat accumulation (3.1‰, 9/2886) were included. Eight were women with a mean age of 47.9 ± 8.0 years old (range, 36–60) (Table 1). Most of them have been infected by blood transfusion (77%, 7/9) and the mean duration of HIV infection was 14.1 ± 5.5 years (range, 6–22). Four PLWH were coinfected with HCV, 1 patient with both HBV and HCV. All the patients have been treated with lamivudine (3TC), among them 7 had a stavudine (d4T) and 2 had a zidovudine (AZT) contained regime, 7 patients received non-nucleoside/nucleotide reverse transcriptase inhibitors (NNRTIs) including nevirapine (NVP) and 2 had efavirenz (EFV). The mean duration for ART was 8.8 ± 2.1 years (range, 6–11). Due to severe toxicity, the Hubei provincial government in 2013 has gradually offered the possibility to replaced d4T by tenofovir (TDF), the only free medicament available in China with reduced metabolic toxicity. At the time of diagnosis of dorsocervical fat accumulation, all patients had limited neck mobility and difficulty to supine before surgery. Their body mass index (BMI) ranged from 18.1 to 28.8 kg/m^2^. The liver radiological examination showed steatohepatitis in 2 patients, whereas cirrhosis has been detected in 1 patient associated with HBV and HCV coinfection. The results of the laboratory examinations revealed a 2 to 3-fold increase of the serum TG over normal values in 8 PLWH (89%), and 1 (11%) had an increased fasting glucose. The mean pre-ART CD4+ T cell count was 91.3 ± 76.5 cells/μL (range, 4–233) and 477.4 ± 271.8 cells/μL (range, 114–926) at the time of surgery.

**Table 1:**
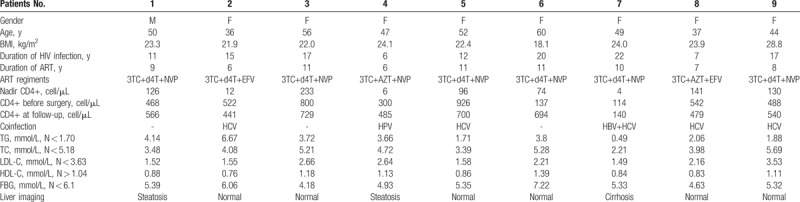
Characteristics of 9 HIV+ patients undergoing excisional lipectomy for dorsocervical lipodystrophy.

### 3.2 Surgical procedures

**Figure 1:**
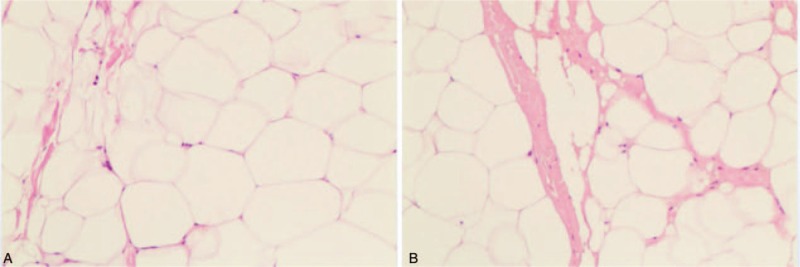
Histologic specimen of abnormal fat from lipodystrophy excision demonstrating fibrous septae and significant variability among adipocytes (A) fibrous septae; (B) adipocytes.

All 9 patients underwent excisional lipectomy of their hypertrophied dorsocervical fat pad. The mean size of the excised specimens was 14 × 11 × 6 cm. There was no clear boundary between aggregated fat and surrounding adipose tissue in all patients. Pathological examination did not show capsule or pseudo-capsule, the tissue was composed of fibrosclerotic zones and significant variation in the shapes and sizes of adipocytes (Fig. 1). The majority of patients (7/9, 78%) underwent surgery without complications and recovered after surgery, the mean duration of hospitalization was 23.4 ± 14.3 days (range, 7–54). Whereas 2 patients (2/9, 22%) underwent additional procedures, including one with seroma complicated by infection and another with wound dehiscence. Patient with seroma were managed by aspiration and drain placement, whereas delayed wound healing was treated by daily dressing changes. For the patient with wound dehiscence, resuturing had been done at the 9th day after surgery. In these 2 patients, intravenous infusion of antibiotic was given. The median follow-up time was 24 months (range, 2–60 months), all 9 patients reported satisfaction with their results, and no recurrences have been observed (Table 2), with the mean CD4+ T cell count was 536 ± 142.8 cells/μL (range, 140–779) at the time of follow-up (Table 1) whereas the HIVRNA viral load were all under detectable level (<20 copies/mL).

**Table 2:**
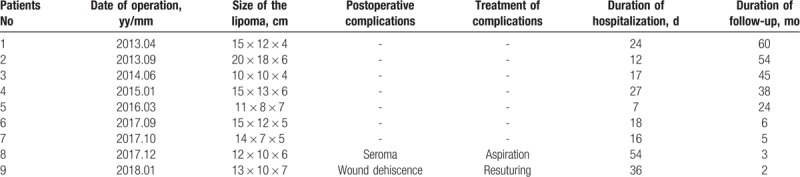
Surgical complications and the follow-up of HIV+ patients undergoing excisional lipectomy for dorsocervical fat pad.

## 4 Discussion

As one of the manifestations of HALS, dorsocervical fat accumulation called “buffalo hump” can become painful with lager size, leading to restricted neck movement, and can be associated with abdomen fat accumulation leading to sleep apnea.^[5]^ Stigma and discrimination, may lead to low adherence to the treatment, accompanying with depression and social withdraw.^[6]^ Several studies have attempted to better understand the pathogenesis, yet the precise mechanism of HALS has not been well determined. However, several factors have been identified which include HIV infection, ART, the genetic background, and host metabolic factors.^[7–9]^ The development and severity of HALS have been initially associated with the duration of HIV infection, and low CD4+ T cell count.^[10,11]^ More recently, Lo et al^[12]^ first reported on contribution of cumulative exposure to ART as one of the strongest factors for dyslipidemia, especially with protease inhibitors (PIs). Whereas now it is well established that each class of drugs has a different effect on fat metabolism, even drugs of the same class may have different effects. In addition, combination of certain drugs acts synergistically to cause dyslipidemia.^[13–15]^ For nucleoside/nucleotide reverse transcriptase inhibitors (NRTIs), dyslipidemia has been associated with exposure to stavudine, zidovudine, and abacavir, whereas tenofovir and lamivudine showed minimal or even a favorable lipid influence.^[16]^ In the NNRTIs, efavirenz has been associated with increases in TC and TG, whereas rilpivirine has been shown to have less effect on lipids compared with efavirenz.^[17]^ Among PIs, lopinavir, ritonavir, and nelfinavir have been associated with lipo-accumulation whereas atazanavir has limited effect.^[18]^ The integrase inhibitors (INIs) class have a little or no effect on lipid, which makes it one of the best choices for patients. Most of our patients had been treated by the combination of 2 NRTIs and 1 NNRTI drugs and no PIs have been used, as recommended by the Chinese therapeutic guideline proposed for first-line choice regimen. Our findings further revealed that patients with dorsocervical fat pad under NRTIs and NNRTI regimens had a significantly greater increased plasma levels of TG than TC. The relationship between changes in body fat and lipid levels is not yet well understood because alterations in the lipid profile do not clearly correlate with body tissue fat deposition. Proposed hypotheses including local effects of increased glucocorticoid production, inhibition of adipocyte differentiation, impaired activity of glucose transporters, mitochondrial toxicity, and more recently the effect of gut dysbiosis.^[19–22]^ In recent years, as the number of HIV infections continues to increase and the duration of ART prolonged, the utilization of PIs (i.e., Kaletra) as the second-line treatment in China is increasing. Apart of the morphologic changes, accompanied cardiovascular diseases in this population may also increase, and prevention of these conditions has become a concern. At the same time, other classes of antiretroviral drugs such as integrase inhibitors, which has been proposed by World Health Organization (WHO) and has been widely used in western countries should be integrated as first-line treatment in China.

Interestingly, we noticed that even more male PLWH were initially included in our study (male: female = 2.5:1), however, in the group of patients with reported “buffalo hump” underwent surgery, there were far more women than men (male: female = 1:8), whereas the patients were mainly men in other studies.^[23–25]^ Is the women more prone to “buffalo hump” formation than men? We think one of the reasons for this discrepancy is the lack of consensus on diagnostic criteria of HALS.^[26]^ The diagnosis is usually based on the patient's self-report of regional body fat changes and physical examination. But it is obviously that an individual's awareness and acceptance of body fat gain may be different. For example, an obese person may not be as concerned with increasing central adiposity, in contrast the women maybe more conscious to their body image and more eagerly to seek for medical attention. In the literature, the formation of “buffalo hump” was merely associated with the infection of HIV. Since in our study, 7 out of the 9 patients have been infected by blood transfusion, 1 patient has been coinfected with HBV whereas the HBVDNA was negative as a result of ART, but 5 out of 9 patients has been coinfected with HCV, which were not the case in other studies, so, does HCV play a synergistic role with HIV in the formation of “buffalo hump,” or especially has an influence on the women should be exploited further.

A study including 72 patients from the Asia-Pacific region showed that the patients receiving ART for >3.8 years had a significantly higher prevalence of HALS than those receiving ART for <3.8 years (16% vs 5%; odds ratio [OR], 4.01; *P* = .001).^[2]^ In an Indian cross-sectional study, the mean duration of ART was 5 years for the development of fat redistribution group.^[27]^ In our study, the average time of HIV infection was 14.1 ± 5.5 years (range, 6–22) and the average time of duration for antiretroviral therapy was 8.8 ± 2.1 years (range, 6–11), which was much longer than the other studies. Tracking the causes, we found that since it remains difficult to accurately define HALS, many clinicians did not make an early diagnosis, and some patients may have been treated surgically late. Hence, in resource-limited regions, apart from ART switching and lifestyle change strategies, the surgical treatment in patients with dorsocervical fat pad are still the cornerstone of the management.^[24,25,28]^ Although the cosmetic and functional results of the surgery are often fulfilled, the main drawback is recurrence of the fat accumulation, with a reported relapse rate up to 50% over a follow up of 12 to 30 months.^[29]^ In our study, during a median of 24 months (range, 2–60) of follow-up, all 9 patients reported satisfaction with their results, there have been no recurrences reported, and the CD4+T cell count were stable for all the patients at the time of follow-up with the viral load under detectable level. This may benefit from drug switch from d4T to TDF. Additionally, our relatively short follow-up time of certain patients could not be ignored, so the long-term effect of surgery needs further observation.

Although 2 surgical options for the treatment of dorsocervical fat pad are proposed which including traditional surgical dermolipectomy and non-invasive ultrasound-assisted liposuction. While the “buffalo humps” are usually composed by fat tissue that is hard, with trabecules and fibrous as shown in our study,^[30,31]^ making passage of the lipoplasty cannulas into the region quite difficult, some studies recommended excisional lipectomy in order to maximal remove the fibrous fatty tissue and theoretically reduce the rate of recurrence.^[25,32]^ Taking together, we suggested to use excisional lipectomy as the primary treatment, with liposuction added when necessary for better contouring of the periphery of the excision, especially when the fat pad is >10 cm.

## 5 Conclusion

Globally, corrective surgery used to treat dorsocervical fat accumulations in patients experiencing considerable physical or psychological discomfort shown a favorable effect and can therefore be considered when necessary. Whereas more drugs with favorable lipid profile should be used to PLWH in the country like China and more prevention strategies should be initiated to control the metabolite effect of ART.

## Acknowledgments

The authors thank Prof. Quanyan Liu from the Department of General Surgery and Dr. Yueying Li from the Department of Pathology, Zhongnan Hospital of Wuhan University, for their assistance with this project. The authors thank all study participants. J-PR is the holder of the Louis Lowenstein Chair in Hematology and Oncology, McGill University and William Turner award from the McGill University Health Centre.

## Author contributions

**Conceptualization:** Xi-en Gui, Qian Cao, Jean-Pierre Routy.

**Data curation:** Song Chen, Xi-en Gui, Qian Cao.

**Formal analysis:** Song Chen, Xi-en Gui, Qian Cao, Jean-Pierre Routy.

**Funding acquisition:** Qian Cao.

**Methodology:** Song Chen, Qian Cao, Jean-Pierre Routy.

**Software:** Song Chen.

**Supervision:** Xi-en Gui, Qian Cao, Jean-Pierre Routy.

**Validation:** Song Chen, Xi-en Gui, Qian Cao, Jean-Pierre Routy.

**Writing – original draft:** Song Chen, Qian Cao.

**Writing – review & editing:** Xi-en Gui, Qian Cao, Jean-Pierre Routy.

## References

[1] Grinspoon S Carr A Cardiovascular risk and body-fat abnormalities in HIV-infected adults. N Engl J Med 2005;352:48–62. 15635112 10.1056/NEJMra041811

[2] Han SH Zhou J Saghayam S Prevalence of and risk factors for lipodystrophy among HIV-infected patients receiving combined antiretroviral treatment in the Asia-Pacific region: results from the TREAT Asia HIV Observational Database (TAHOD). Endocr J 2011;58:475–84. 21521929 10.1507/endocrj.k10e-407 PMC3329967

[3] Chen D Misra A Garg A Clinical review 153: lipodystrophy in human immunodeficiency virus-infected patients. J Clin Endocrinol Metab 2002;87:4845–56. 12414837 10.1210/jc.2002-020794

[4] Carr A Cooper DA Images in clinical medicine. Lipodystrophy associated with an HIV-protease inhibitor. N Engl J Med 1998;339:1296. 9791146 10.1056/NEJM199810293391806

[5] Tsui E Bogdasarian R Blomain E The successful use of lipectomy in the management of airway obstruction in a woman with HIV-associated lipodystrophy. BMJ Case Rep 2015;2015:bcr2014208053. 10.1136/bcr-2014-208053 PMC4336867 25694636

[6] Katz IT Ryu AE Onuegbu AG Impact of HIV-related stigma on treatment adherence: systematic review and meta-synthesis. J Int AIDS Soc 2013;16 3 suppl 2:18640. 24242258 10.7448/IAS.16.3.18640 PMC3833107

[7] Sattler FR Pathogenesis and treatment of lipodystrophy: what clinicians need to know. Top HIV Med 2008;16:127–33. 18838747

[8] Sellmeyer DE Grunfeld C Endocrine and metabolic disturbances in human immunodeficiency virus infection and the acquired immune deficiency syndrome. Endocr Rev 1996;17:518–32. 8897023 10.1210/edrv-17-5-518

[9] Raposo MA Armiliato GNA Guimaraes NS Metabolic disorders and cardiovascular risk in people living with HIV/AIDS without the use of antiretroviral therapy. Rev Soc Bras Med Trop 2017;50:598–606. 29160505 10.1590/0037-8682-0258-2017

[10] Seminari E Tinelli C Minoli L Evaluation of the risk factors associated with lipodystrophy development in a cohort of HIV-positive patients. Antivir Ther 2002;7:175–80. 12487384

[11] Miller J Carr A Emery S HIV lipodystrophy: prevalence, severity and correlates of risk in Australia. HIV Med 2003;4:293–301. 12859330 10.1046/j.1468-1293.2003.00159.x

[12] Lo JC Mulligan K Tai VW “Buffalo hump” in men with HIV-1 infection. Lancet 1998;351:867–70. 9525364 10.1016/S0140-6736(97)11443-X

[13] Tsai FJ Cheng CF Lai CH Effect of antiretroviral therapy use and adherence on the risk of hyperlipidemia among HIV-infected patients, in the highly active antiretroviral therapy era. Oncotarget 2017;8:106369–81. 29290955 10.18632/oncotarget.22465 PMC5739740

[14] Maggi P Di Biagio A Rusconi S Cardiovascular risk and dyslipidemia among persons living with HIV: a review. BMC Infect Dis 2017;17:551. 28793863 10.1186/s12879-017-2626-z PMC5550957

[15] Mallewa JE Wilkins E Vilar J HIV-associated lipodystrophy: a review of underlying mechanisms and therapeutic options. J Antimicrob Chemother 2008;62:648–60. 18565973 10.1093/jac/dkn251

[16] Gallant JE Staszewski S Pozniak AL Efficacy and safety of tenofovir DF vs stavudine in combination therapy in antiretroviral-naive patients: a 3-year randomized trial. JAMA 2004;292:191–201. 15249568 10.1001/jama.292.2.191

[17] Molina JM Cahn P Grinsztejn B Rilpivirine versus efavirenz with tenofovir and emtricitabine in treatment-naive adults infected with HIV-1 (ECHO): a phase 3 randomised double-blind active-controlled trial. Lancet 2011;378:238–46. 21763936 10.1016/S0140-6736(11)60936-7

[18] Kim RJ Wilson CG Wabitsch M HIV protease inhibitor-specific alterations in human adipocyte differentiation and metabolism. Obesity (Silver Spring) 2006;14:994–1002. 16861604 10.1038/oby.2006.114

[19] Behrens GM Stoll M Schmidt RE Lipodystrophy syndrome in HIV infection: what is it, what causes it and how can it be managed? Drug Saf 2000;23:57–76. 10915032 10.2165/00002018-200023010-00004

[20] Giralt M Domingo P Villarroya F Adipose tissue biology and HIV-infection. Best Pract Res Clin Endocrinol Metab 2011;25:487–99. 21663842 10.1016/j.beem.2010.12.001

[21] Cereijo R Gallego-Escuredo JM Moure R The molecular signature of HIV-1-associated lipomatosis reveals differential involvement of brown and beige/Brite adipocyte cell lineages. PLoS One 2015;10:e0136571. 26305325 10.1371/journal.pone.0136571 PMC4549259

[22] Non LR Escota GV Powderly WG HIV and its relationship to insulin resistance and lipid abnormalities. Transl Res 2017;183:41–56. 28068521 10.1016/j.trsl.2016.12.007

[23] Piliero PJ Hubbard M King J Use of ultrasonography-assisted liposuction for the treatment of human immunodeficiency virus-associated enlargement of the dorsocervical fat pad. Clin Infect Dis 2003;37:1374–7. 14583872 10.1086/379073

[24] Connolly N Manders E Riddler S Suction-assisted lipectomy for lipodystrophy. AIDS Res Hum Retroviruses 2004;20:813–5. 15320984 10.1089/0889222041725208

[25] Warren AG Borud LJ Excisional lipectomy for HIV-associated cervicodorsal lipodystrophy. Aesthet Surg J 2008;28:147–52. 19083520 10.1016/j.asj.2007.12.003

[26] Carter VM Hoy JF Bailey M The prevalence of lipodystrophy in an ambulant HIV-infected population: it all depends on the definition. HIV Med 2001;2:174–80. 11737398 10.1046/j.1468-1293.2001.00073.x

[27] Kumar NS Shashibhushan J Malappa Lipodystrophy in human immunodeficiency virus (HIV) patients on highly active antiretroviral therapy (HAART). J Clin Diagn Res 2015;9:OC05–8. 10.7860/JCDR/2015/12979.6183 PMC4572985 26393154

[28] Shridharani SM Mohan R A 51-year-old man with HIV and cervicodorsal lipodystrophy (buffalo hump). JAMA 2013;309:1289–90. 23532244 10.1001/jama.2013.2552

[29] Sharma D Bitterly TJ Buffalo hump in HIV patients: surgical management with liposuction. J Plast Reconstr Aesthet Surg 2009;62:946–9. 18468504 10.1016/j.bjps.2007.10.086

[30] del Mar Gutierrez M Mateo G Domingo P Strategies in the treatment of HIV-1-associated adipose redistribution syndromes. Expert Opin Pharmacother 2007;8:1871–84. 17696790 10.1517/14656566.8.12.1871

[31] Mateo MG Gutierrez MdM Vidal F An update on the pharmacological strategies in the treatment of HIV-1-associated adipose redistribution syndromes. Expert Opin Pharmacother 2014;15:1749–60. 24934336 10.1517/14656566.2014.928694

[32] Gervasoni C Ridolfo AL Vaccarezza M Long-term efficacy of the surgical treatment of buffalohump in patients continuing antiretroviral therapy. AIDS 2004;18:574–6. 15090816 10.1097/00002030-200402200-00030

